# Inflammatory state moderates response to cannabis on negative affect and sleep quality in individuals with anxiety

**DOI:** 10.3389/fnbeh.2025.1549311

**Published:** 2025-07-01

**Authors:** Jonathon K. Lisano, Carillon J. Skrzynski, Gregory Giordano, Angela D. Bryan, L. Cinnamon Bidwell

**Affiliations:** ^1^Institute of Cognitive Science, University of Colorado Boulder, Boulder, CO, United States; ^2^Department of Psychology and Neuroscience, University of Colorado Boulder, Boulder, CO, United States

**Keywords:** THC, CBD, cytokines, depression, anxiety, stress

## Abstract

**Introduction:**

Inflammation has been implicated as an underlying pathology in negative affect and sleep disruption. Cannabinoids like delta-9-tetrahydrocannabinol (THC) and cannabidiol (CBD) have demonstrated anti-inflammatory properties. This study aimed to assess if cannabis use altered cytokine concentration and whether inflammatory status moderated the influence of 4 weeks of cannabis use on negative affect and sleep quality in anxious individuals.

**Methods:**

Participants with mild or greater anxiety (*n* = 147) were assigned to one of three cannabis chemovars (THC + CBD, THC, CBD), asked to consume their products *ad libitum* for 4 weeks, and were compared to a group of participants with anxiety who did not use cannabis (*n* = 24). Measures of negative affect (Depression Anxiety and Stress Scale-21: DASS-21), sleep quality (Pittsburgh Sleep Quality Index: PSQI), and plasma cytokine concentrations were measured at Baseline and Week-4. Multilevel modeling assessed if there were group-dependent changes in cytokine concentrations over time, and whether baseline inflammation moderated the association between cannabis use and both negative affect and sleep quality.

**Results:**

There were no group-dependent changes in cytokine concentrations throughout the study (*p* = 0.12). It was observed that baseline inflammatory state moderated the group-by-time relationship for DASS-21 (*p* < 0.001) and PSQI (*p* = 0.04). In both models, chemovars higher in CBD produced more consistent improvements, while THC-associated improvements varied by baseline inflammatory state.

**Conclusion:**

These novel findings suggest that baseline inflammatory status influences the relationship between cannabis use, negative affect, and sleep quality in people with anxiety.

## Introduction

It is currently estimated that 29.57% of adults in the United States (US) suffer from mild to moderate anxiety, making it one of the most prevalent mental health conditions (Mahmud et al., 2023). Anxiety is a core component of negative affect and is broadly correlated with other components of negative affect like depression and stress. Not only is anxiety itself a pernicious health problem, but it may also perturb other negative health outcomes, including insomnia and poor sleep quality. Indeed, 60%–70% of individuals diagnosed with Generalized Anxiety Disorder (GAD) report poor sleep quality (Papadimitriou and Linkowski, 2005), and individuals with any prior anxiety disorder are at higher risk for insomnia and other sleep disturbances (Johnson et al., 2006). Thus, investigation into potential treatments for co-morbid anxiety and sleep is crucial. Unfortunately, when patients present with both anxiety and sleep disruption, treatment often focuses on either anxiety or sleep disruption, rather than both simultaneously, with expectations that symptoms of the other will improve as the primary treatment progresses (Mason et al., 2023; Morin et al., 2023). As such, research into methods of addressing both anxiety and sleep disturbance and underlying factors that affect the efficacy of these methods, are indicated.

Inflammation is an often overlooked, yet complex, underlying pathology that has been implicated in both negative affect and sleep disruption. Specifically, increased levels of cytokines, inflammatory proteins released from immune cells and stressed tissues, have been associated with increased anxiety (Hoge et al., 2009; Vieira et al., 2010; Gualtieri et al., 2020) and decreased sleep quality (Coksevim et al., 2018; Abd El-Kader and Al-Jiffri, 2019; Lee et al., 2021). Despite these associations, to our knowledge, only one study to date has investigated inflammation in the context of mitigating anxiety. Johnson et al. (2021) found that cytokine concentrations did not moderate the effects of cognitive behavioral therapy or metacognitive therapy treatment for anxiety, though the study had a small sample size, did not assess changes in sleep quality in parallel with anxiety, nor did it use any pharmacological intervention with purported anti-inflammatory effects (Johnson et al., 2021). The relationship between inflammation and depression is more established. Research suggests an interconnection with co-progression in the counterpart if the other increases (Berk et al., 2013; Lee and Giuliani, 2019). Additionally, anti-inflammatory treatments have shown promise in reducing depressive symptoms (Bai et al., 2020; Faridhosseini et al., 2014).

Emerging research indicates that cannabinoids like delta-9-tetrahydrocannabinol (THC) and cannabidiol (CBD) found within the *Cannabis Sativa* plant have anti-inflammatory properties (Kozela et al., 2010; Henshaw et al., 2021). Given the association between negative affect, poor sleep quality, and inflammation, it is perhaps not surprising that 70.7 and 37.8% of medical cannabis users report using cannabis products to improve sleep and relieve anxiety, respectively (Reinarman et al., 2011). Empirically, previous research from the data set used in the current paper (Bidwell et al., 2023; Bidwell et al., 2024) and from other labs (Tervo-Clemmens et al., 2023; Ried et al., 2023; Walsh et al., 2021) have demonstrated improvements in both sleep and anxiety with cannabis use. Existing literature seems to suggest that higher amounts of CBD are associated with greater improvements (Bidwell et al., 2023; Bidwell et al., 2024). However, this may depend on the amount and frequency of use as other evidence suggests higher quantity of cannabis and using it more often are associated with worsened sleep and anxiety (Winiger et al., 2022).

While the impact of THC versus CBD on anxiety has been previously explored by our research group (Bidwell et al., 2024), this study expands on these findings, exploring the impact of circulating cytokine concentration on negative affect and sleep quality in participants utilizing various cannabis chemovars. Given the linkages between anxiety, sleep, and inflammation two possible mechanistic pathways might be examined. First, it is possible that cannabis-induced changes in inflammatory cytokine concentrations during 4-weeks of *ad libitum* use of one of three cannabis flower chemovars (THC + CBD [containing equal amounts of THC and CBD], THC-dominant, CBD-dominant) might lead to changes in negative affect and sleep (cytokine levels *mediate* the association among cannabis and sleep). In that case, we would predict based on prior studies (Kozela et al., 2010; Henshaw et al., 2021) that the THC + CBD and CBD chemovars would produce more pronounced reductions in circulating cytokines than the THC chemovar. The second possibility is that individual differences in inflammation (i.e., circulating cytokine concentrations at study entry) might *moderate* associations between cannabis use and both negative affect and sleep outcomes. If this is the case, we hypothesized that changes in anxiety and subjective sleep quality throughout the 4-week intervention would be dependent on both cannabis chemovar and inflammatory status, with products higher in CBD (i.e., THC + CBD, and CBD-dominant) leading to greater improvements at higher levels of inflammation compared to the THC-dominant chemovar. In each case, the effects will be compared to participants with anxiety who did not use any cannabis over the same time frame.

## Materials and methods

### Participants

**FIGURE 1:**
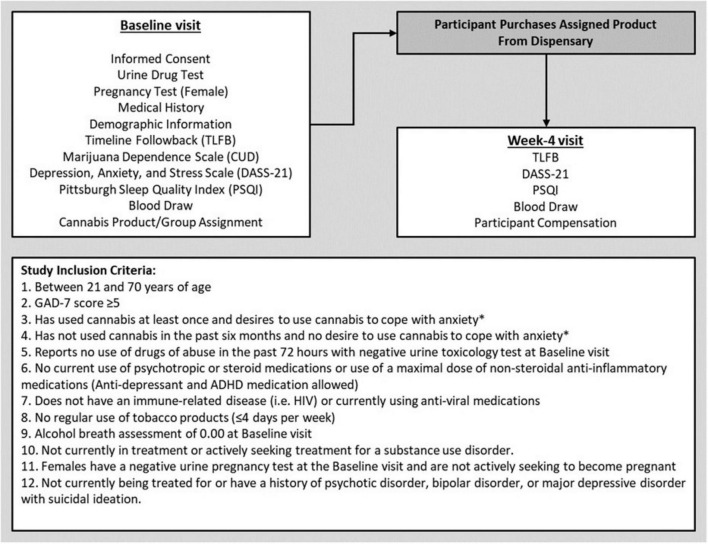
Study visit outline and participant inclusion criteria. *Participants had to meet only one of these criteria to participate in the study.

A subsample from a larger, longitudinal study [preregistered at ClinicalTrials.gov (NCT03491384)] observing the effects of cannabis use in individuals with anxiety was used for this analysis. The study followed all ethical standards for human participants outlined in the Declaration of Helsinki and was approved by the Institutional Review Board (IRB) of the University of Colorado, Boulder. Out of the original sample of 300, a total of 171 participants were included in the present analysis based on available inflammatory data measured via concentrations of circulating cytokines (Participant Consort: Supplementary Figure 1). All participants were recruited from the local Boulder/Denver metropolitan area utilizing posters, social media, mailed flyers, and local presentations at community events. Participants were eligible if they were of legal age for cannabis use (i.e., 21+ years of age) and reported at least mild anxiety indicated by a score ≥ 5 on the Generalized Anxiety Disorder-7 scale (GAD-7) (Spitzer et al., 2006). Additional inclusion criteria can be found in Figure 1.

### Cannabis group assignment

Participants in the cannabis use group were randomly assigned to one of three distinct cannabis flower chemovar product groups [THC + CBD (12% THC, 12% CBD); THC (24% THC; < 1% CBD); or CBD (< 1% THC; 24% CBD)] during their Baseline visit. This randomization was based on a random number table generated by the study statistician at the start of the study. Product concentrations of THC and CBD were based on cannabis flower products commercially available in Colorado to maximize external validity. Each product was labeled consistent with requirements by the state of Colorado following testing in an International Organization of Standards (ISO) 17025 accredited laboratory. The assigned study products were purchased directly by participants at a local partner dispensary. This partner dispensary had no role in funding, study design, data collection, analysis, interpretation, or writing of this report. To cross-validate that participants purchased the product that they were assigned to, they were asked to submit a photo of the product label via the REDCap (Research electronic data capture) application (Harris et al., 2009). This photo was reviewed and validated by study staff who were not involved with in-person data collection to maintain researcher blinding. Participants were asked to refrain from using any non-study-assigned cannabis products while enrolled in the study and were given safety instructions consistent with materials provided by the Colorado Marijuana Enforcement Division. To facilitate *ad libitum* use of their product concerning timing, frequency, and amount used, participants were free to purchase as much or as little of the study product as they needed to fit their natural use patterns. Participants in the non-use group were asked to refrain from using any cannabis products for the duration of the 4-week study.

#### Cannabis product safety and monitoring

While participants assigned to the THC + CBD, THC, and CBD groups were required to have previous experience with using cannabis (see inclusion criteria), it was still possible that some participants might have experienced adverse effects from their study-assigned product such as changes in mood/affect, sleepiness, paranoia, increased heart rate, or dependence. As a result, participants were monitored during the study period by a licensed clinical psychologist to monitor for clinically significant events. Increases in anxiety, adverse events, and discomfort were monitored, and if clinically severe or impairing anxiety occurred, the participants would be removed from the study and referred to appropriate mental health treatment. No such clinically significant events occurred during the course of the study.

### Study visits and outline

Data for these analyses were collected over a 4-week period, consisting of two in-person visits with research staff. The Baseline visit lasted approximately 2.5 h, and participants were compensated $80 cash for their time and participation. The second study visit (Week-4) was completed 4 weeks after Baseline. The Week-4 visit was conducted in our mobile pharmacology laboratory which was driven to the participant’s place of residence. At the end of the Week-4 visit, participants were compensated $80 for their time. A complete outline of study procedures and measures collected at each visit can be found in Figure 1.

### Measures

During their Baseline visit participants completed informed consent, a medical history questionnaire, provided demographic information, and verified inclusion eligibility information. All surveys and questionnaires were administered electronically through the REDCap application. Participants were also instructed to refrain from using cannabis for 24 h before their Baseline and Week-4 study visits.

#### Cannabis use disorder and frequency of use

The frequency, method, amount, and form of recent cannabis use was measured using an online version of the Timeline Followback assessment [O-TLFB; (Martin-Willett et al., 2020)]. Participants were assessed for cannabis use disorder (CUD) symptoms utilizing the Marijuana Dependence Scale (α = 0.80), an 11-item questionnaire modified for DSM-5 criteria (Callahan et al., 2013; Lozano et al., 2006).

#### Depression, anxiety, and stress

The Depression Anxiety and Stress Scale-21 (DASS-21) (Henry and Crawford, 2005) is a validated clinical measure used to assess an individual’s negative affective state. The 21 items of the DASS-21 are subdivided into three 7-item subscales to measure depression (α = 0.89), anxiety (α = 0.64), and stress (α = 0.81). These three subscales can be analyzed individually or combined to create a composite score (α = 0.89).

#### Subjective sleep quality

The Pittsburgh Sleep Quality Index (PSQI; α = 0.70) is a 19-item self-report questionnaire (Buysse et al., 1989). Participants rate their sleep quality, sleep latency, habitual sleep efficiency, sleep disturbances, use of sleep medication, and daytime dysfunction. These components are used to calculate a cumulative PSQI score with increasing scores indicating poorer subjective sleep quality. Clinically, a PSQI score > 5 is defined as having poor sleep quality.

#### Self-report diet and activity status

##### Diet

A single item asking, “In general, how healthy is your overall diet?” had participants self-rate their diet using a 1 to 5 scale (Subar et al., 2001). With “1” being poor and “5” being excellent. This self-report measure has been previously validated and found to significantly correlate with more comprehensive dietary recall measures like the Healthy Eating Index-2010 (Adjoian et al., 2016).

##### Activity

The Stanford Leisure-Time Activity Categorical Item (L-Cat) is a single item asking, “During the past 2 weeks, which statement best describes the kind of physical activity you usually did? Do not include the time you spent working a job.” Participants self-rated their activity status on a scale of 1 to 6 (Kiernan et al., 2013). A response of “1” indicated not engaging in physical activity more than once or twice per month, with increasing levels of activity as responses moved toward “6” which indicated engaging in daily activity for at least 30 min, i.e., riding hard on a bike or running.

#### Blood collection, analysis of cannabinoid content, and cytokine concentration

##### Blood collection

Blood was collected at the Baseline and Week-4 study visits utilizing standard phlebotomy procedures. A trained phlebotomist collected 20 mL of blood in EDTA-treated vacutainers. These vacutainers were then centrifuged at 1000× *g* for 10 min in a Sorvall Legend X1 (Thermofisher; Waltham, MA). Following centrifugation, plasma was harvested and aliquoted in microcentrifuge tubes. Samples were stored at −80°C until analysis.

##### Blood cannabinoids

To help verify participant chemovar adherence, plasma samples collected at the Week-4 study visit were assessed for Delat-9-tetrahydrocannabinol (THC), 11-Hydroxy- tetrahydrocannabinol (11-OH-THC), and cannabidiol (CBD) concentration. Plasma cannabinoid analysis was performed by the iC42 laboratory at the University of Colorado Anschutz School of Medicine using a validated high-performance liquid chromatography/mass-spectroscopy (HPLC-MS/MS) protocol (API550034) (Klawitter et al., 2017).

##### Circulating cytokines

Circulating concentrations of cytokines interleukin 1a (IL-1a), 1b (IL-1b), 6 (IL-6), 8 (IL-8), 12 (IL-12), and tumor necrosis factor-α (TNFα) were quantified from 10 mL of plasma collected during the Baseline and Week-4 visits. Due to the acquisition of Aushon Biosystems Inc., by Quanterix Corp. midstudy, and the transition to a new legacy kit, cytokines were analyzed via the Ciraplex Human Cytokine 10-plex Array (Aushon Biosystems Inc., Billerica, MA), Ciraplex Human Cytokine 10-plex Array (Quanterix Corp. Billerica, MA), or the Ciraplex Human Cytokine 7-plex Legacy Array (Quanterix Corp. Billerica, MA). Samples were run in duplicate, and all assays were prepared per the manufacturer’s instructions. Inter-assay variation was less than 15% and intra-assay variation was less than 10%. Cytokine values below the limit of quantification (LLOQ) were replaced with LLOQ/2. To reduce variability, concentrations of IL-1a, IL-1b, IL-6, IL-8, IL-12, and TNFα greater than 3 standard deviations (SD) from the mean were Winsorized to mean + 3 SD. Concentrations of the cytokines IL-1a, IL-1b, IL-6, IL-8, IL-12, and TNFα, which are generally regarded as proinflammatory, were highly correlated with each other (r’s = 0.78–0.96). As a result of their high degree of correlation and to better understand how global concentrations of circulating pro-inflammatory cytokines impact the outcomes, z-scores for each cytokine were calculated. Those scores were averaged across all cytokines at Baseline and Week-4 (Davis et al., 2010; Manigault et al., 2021). This average proinflammatory cytokine z-score was then used in all statistical models. Note. There were no differences in cytokine z-scores between the three assays used for cytokine analysis.

### Statistical analysis

All statistical analyses were conducted in R (R Core Team, 2020) utilizing the dplyr (Wickham et al., 2023), sjplot (Lüdecke, 2023), ltm (Rizopoulos, 2022), emmeans (Lenth, 2023), and gee (Carey et al., 2023) libraries. All figures were generated in R using the ggplot2 (Wickham et al., 2024) library. Correlations among primary descriptive and outcome variables (Supplementary Figure 2) were estimated to assess relationships and identify potential covariates for inferential statistical models. Note. The models described below could not control for blood cannabinoid concentration or frequency of study product use. This is due to the non-use group not engaging in any cannabis use (i.e., blood THC/CBD and frequency of cannabis use being zero for these individuals), and if included these variables would have skewed the analyses.

#### *Post hoc* power analysis

*Post hoc* power analyses were conducted with G*power (analysis of variance repeated measures within-between interaction) (Faul et al., 2007) and indicated that a power level of 0.85 can be achieved with a Cohen’s f as small as 0.17 (partial η^2^ of 0.03) (Cohen, 2013), 171 participants in four groups with two timepoints, and correlation between timepoints, sphericity assumptions, and *p*-values as low as 0.05, 0.50, and 0.005, respectively. While *post hoc* power analyses are generally not recommended (Zhang et al., 2019), these findings should be considered within this context.

#### Cytokine concentration over time

To address whether cannabis use was associated with changes in inflammation over time, a generalized estimating equations (GEE) model, assessing time (Baseline, Week-4), group (Non-Use, THC + CBD, THC, CBD), and the group by time interaction was run due to non-normal distribution of the average cytokine z-score. Age, body mass index (BMI), diet, activity status, and time of day when blood was collected were utilized as covariates due to previously established relationships between these variables and cytokine concentrations. These analyses showed no significant changes in cytokine concentration over time for any group (see section “Results”), so no mediational analyses were conducted.

#### Moderation models for DASS-21 and PSQI

Previous research on negative affect has observed that inflammatory status moderated risk of developing depression in female breast cancer survivors (Manigault et al., 2021). To extend the data, similar models were run to assess whether Baseline cytokines moderate group-dependent (Non-Use, THC + CBD, THC, CBD) changes over time (Baseline to Week-4) in negative affect (DASS total) and subjective sleep quality (PSQI). As a result, two GEE models were run due to the non-normal distribution of DASS and PSQI scores. In these models, cytokine concentrations at Baseline were utilized as a predictor in addition to participant group and time, as well as all two- and three-way interactions (e.g., time × group, time × cytokine, cytokine × group, time × cytokine × group). Models controlled for participant age, BMI, diet, activity status, and time of day when blood was collected. Where interactions were significant, tests of simple effects were conducted. Secondary models utilizing GEE were performed for the three (depression, anxiety, stress) DASS-21 subscales using the same approach described. In models where interactions with Baseline cytokine concentrations were significant, the emmeans library was used to calculate estimated means by group by time at low (mean – 1 SD), average (mean), and high (mean + 1 SD) cytokine concentrations (Bauer and Curran, 2005).

## Results

### Descriptive information

A total of 171 participants completed study measures at the Baseline and Week-4 visits and had cytokine data available for analysis. Of the 171, 24 participants were in the group that did not use cannabis during the study, 51 were in the THC + CBD group, 44 were in the THC group, and 52 were in the CBD group. There were no differences in age, BMI, or GAD-7 score at Baseline among the four groups. Detailed participant characteristics can be found in Table 1. Scores for CUD at Baseline and the frequency of study product use by group throughout the 4-week study can be found in Table 1. Within the three cannabis use groups (THC + CBD, THC, CBD) there were no differences in CUD at Baseline (*F*(2,141) = 0.33, *p* = 0.72, and no significant difference in how frequently participants used their assigned study product throughout the study [*F*(1,140) = 1.31, *p* = 0.25)].

**TABLE 1:** Participant characteristics.

	Overall sample (*N* = 171)	Non-use (*n* = 24)	THC + CBD (*n* = 51)	THC (*n* = 44)	CBD (*n* = 52)
Demographics					
Age	31.35 ± 12.32	31.75 ± 11.83	31.71 ± 12.66	32.70 ± 13.40	29.67 ± 11.39
Gender [no. (%) female]	94 (55.0%)	16 (66.7%)	26 (51.0%)	23 (52.3%)	29 (55.8%)
BMI (kg/m^2^)	24.01 ± 4.58	25.21 ± 4.45	24.38 ± 4.24	23.54 ± 4.75	23.49 ± 4.81
Education [no. (%) bachelors or higher]	105 (61.4%)	21 (87.5%)	28 (54.9%)	26 (59.1%)	30 (57.7%)
Ethnicity [no. (%)]					
American Indian or Alaska native	3 (1.8%)	1 (4.2%)	2 (3.9%)	0 (0.0%)	0 (0.0%)
Asian	7 (4.1%)	2 (8.3%)	2 (3.9%)	1 (2.3%)	2 (3.8%)
African American or Black	4 (2.3%)	0 (0.0%)	2 (3.9%)	2 (4.5%)	0 (0.0%)
Hispanic or Latino	13 (7.6%)	2 (8.3%)	4 (7.8%)	3 (6.8%)	4 (7.7%)
More than one race/ethnicity	4 (2.3%)	0 (0.0%)	2 (3.9%)	2 (4.5%)	0 (0.0%)
White	135 (78.9%)	18 (75.0%)	38 (74.5%)	35 (79.5%)	44 (84.6%)
Prefer not to answer	5 (2.9%)	1 (4.2%)	1 (1.9%)	1 (2.3%)	2 (3.8%)
GAD-7 score	11.81 ± 4.05	10.71 ± 3.94	11.87 ± 3.52	11.52 ± 4.41	12.49 ± 4.26
Baseline cytokine concentration (pg/mL)	194.5 ± 330.7	151.8 ± 197.3	211.9 ± 397.3	151.4 ± 189.1	232.1 ± 394.0
Cannabis use disorder symptoms (CUD)	1.78 ± 2.29	0.00 ± 0.00	2.10 ± 2.63	2.25 ± 2.21	1.86 ± 2.17
Frequency of study product use (4-week study period)	12.04 ± 8.93	0.00 ± 0.00	14.24 ± 7.86	15.54 ± 8.41	12.46 ± 7.87
Days of any alcohol use (past 14 days)	3.68 ± 3.56	4.29 ± 4.25	3.81 ± 3.70	3.16 ± 3.32	3.72 ± 3.32

Baseline cytokine concentration is the summed concentration of cytokines IL-1a, IL-1b, IL-6, IL-8, IL-12, and TNF-a. These raw cytokine values were not used in the analyses (see “circulating cytokines”).

### Blood cannabinoid concentration

At the Week-4 visit, the cumulative concentration of THC + 11-OH-THC in the blood was highest in the THC group (Supplementary Figure 3). Similarly, concentrations of CBD in the blood were highest in the CBD group (Supplementary Figure 3). There were no detectable concentrations of THC, 11-OH-THC, or CBD in samples of the group that did not use cannabis. Due to the 24-h cannabis abstinence period before each of the visits, the blood cannabinoid values presented are representative of stable values and not representative of concentrations post-acute use.

### Change in cytokine concentration

The GEE analysis did not show a significant effect of time (*z* = −1.32, *p* = 0.19) or group (z = −1.55, *p* = 0.12), nor was there a significant group-by-time interaction (*z* = 1.54, *p* = 0.12). Average cytokine concentration z-score did not change over the 4 weeks of the study for any group. Thus, changes in cytokines as a mediator of the effects of cannabis on DASS and PSQI outcomes were not examined further.

### Baseline cytokine moderation models for DASS

**FIGURE 2:**
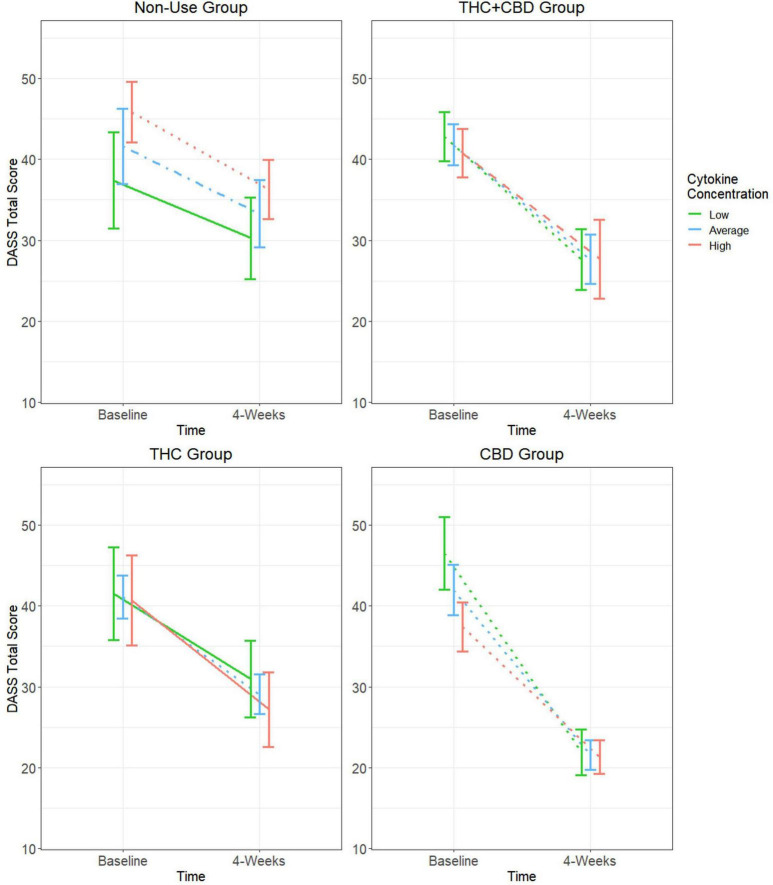
Moderating effect of baseline cytokine concentration on the relationship between cannabis use group and changes in DASS total score. Group estimated means ± SE are plotted. *P*-values of simple effects are represented by the following: solid *p* > 0.05, dashed *p* < 0.05, dot-dash *p* < 0.01, dotted *p* < 0.001. Age, body mass index (BMI), diet, activity status, and time of day when blood was collected were used as covariates in this moderation model.

Consistent with prior analyses (Bidwell et al., 2024), GEE analysis showed a main effect of time with decreases in DASS total scores from Baseline to Week-4 (Table 2). This analysis also revealed a significant group-by-time-by-cytokine concentration three-way interaction (Figure 2 and Table 2 and). Tests of simple effects in the THC + CBD group showed significant decreases across time at low (difference = −15.20, SE = 3.5, *p* < 0.001), average (difference = −14.13, SE = 3.0, *p* < 0.001), and high (difference = −13.07, SE = 3.95, *p* = 0.01) cytokine concentrations. Similar effects were observed in the CBD group at low (difference = −24.62, SE = 3.5, *p* < 0.001), average (difference = −20.38, SE = 2.6, *p* < 0.001), and high (difference = −16.15, SE = 1.9, *p* < 0.001) cytokine concentrations. In the THC group, significant decreases in DASS total over time were observed at average (difference = −12.03, SE = 2.9, *p* < 0.001) but not low or high cytokine concentrations (*p’s* > 0.05). Participants who did not use cannabis experienced significant decreases in DASS total at average (difference = −8.35, SE = 2.5, *p* = 0.01) and high (difference = −9.56, SE = 2.4, *p* < 0.001) but not low (*p* > 0.05) cytokine concentrations.

**TABLE 2:** Generalized estimating equation models predicting outcomes from time, group, cytokine concentration, time by group, time by cytokine concentration, and time by group by cytokine concentration.

	DASS total		DASS depression		DASS anxiety		DASS stress		PSQI	
Predictors	Z score	*P*-value	Z score	*P*-value	Z score	*P*-value	Z score	*P*-value	Z score	*P*-value
Time	−3.40	<0.001	−1.74	0.08	−1.55	0.12	−4.20	<0.001	0.32	0.75
Group	0.93	0.35	0.60	0.55	2.19	0.03	−0.83	0.41	3.25	0.001
Cytokines	2.50	0.01	1.20	0.23	2.26	0.02	2.67	0.007	−1.18	0.24
Time × group	−3.39	<0.001	−2.53	0.01	−3.61	<0.001	−2.64	0.008	−2.87	<0.01
Time × cytokines	−1.24	0.21	−0.50	0.62	−0.26	0.79	−1.72	0.09	1.02	0.31
Time × group × cytokines	3.53	<0.001	1.99	0.04	4.59	<0.001	1.81	0.07	−2.09	0.04

Cytokines = Average of the z-score adjusted plasma concentrations of the cytokines interleukin 1a (IL-1a), 1b (IL-1b), 6 (IL-6), 8 (IL-8), 12 (IL-12), and tumor necrosis factor-α (TNFα).

Due to significant decreases in negative affect in participants who did not use cannabis, comparisons of the rate of change over time between each of the cannabis use groups and the cannabis non-use group were performed. In the CBD group, it was observed that DASS total scores decreased at a significantly greater rate at low (difference = −17.50, SE = 4.6, *p* < 0.001), average (difference = −12.00, SE = 3.6, *p* < 0.001), and high (difference = −6.59, SE = 3.0, *p* = 0.03) cytokine concentrations when compared to those not using cannabis products. These effects were not observed at any cytokine concentration when making similar comparisons of the cannabis non-use group to the THC + CBD and THC groups (*p*’s > 0.05).

#### DASS subscales: depression, anxiety, and stress

Among the three DASS subscales, there were significant three-way interactions for DASS depression and anxiety but not for DASS stress (Table 2). For DASS depression, the CBD group experienced significant decreases over time at low (difference = −7.56, SE = 1.8, *p* < 0.001), average (difference = −6.49, SE = 1.3, *p* < 0.001), and high (difference = −5.41, SE = 0.9, *p* < 0.001) cytokine concentrations (Supplementary Figure 4A). Within the THC + CBD and THC groups, significant decreases in DASS depression over time were only observed at average cytokine concentrations (THC + CBD: difference = −4.33, SE = 1.4, *p* = 0.02; THC: difference = −4.40, SE = 1.4, *p* = 0.03). There were no significant changes in DASS depression at any cytokine concentration in the cannabis non-use group (*p*’s > 0.05). For DASS anxiety, the THC + CBD and CBD groups experienced significant decreases over time at low (THC + CBD: difference = −4.27, SE = 1.0, *p* < 0.001; CBD: difference = −8.09, SE = 1.2, *p* < 0.001), average (THC + CBD: difference = −3.81, SE = 0.9, *p* < 0.001; CBD: difference = −5.79, SE = 0.9, *p* < 0.001), and high (THC + CBD: difference = −3.35, SE = 1.1, *p* = 0.02; CBD: difference = −3.49, SE = 0.7, *p* < 0.001) baseline cytokine concentrations (Supplementary Figure 4B). Same as DASS depression, the THC group only experienced significant decreases in DASS anxiety at average cytokine concentrations (difference = −3.32, SE = 0.8, *p* = 0.001). There were no significant changes in DASS anxiety at any cytokine concentration within the cannabis non-use group (*p*’s > 0.05).

### Baseline cytokine moderation models for PSQI

**FIGURE 3:**
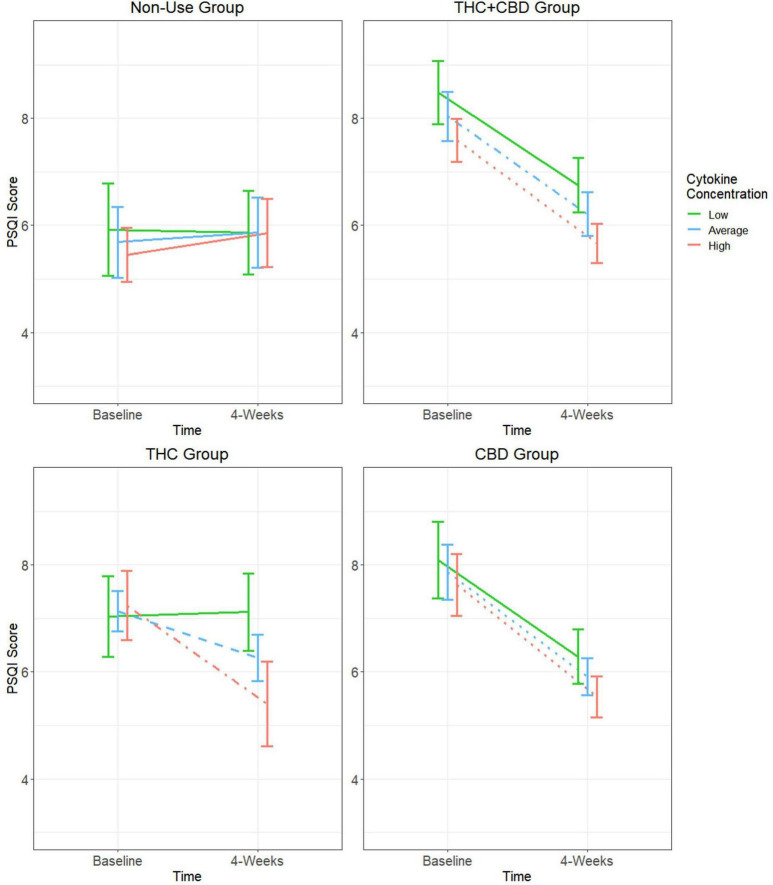
Moderating effect of baseline cytokine concentration on the relationship between cannabis use group and changes in PSQI score. Group estimated means ± SE are plotted. *P*-values of simple effects are represented by the following: solid *p* > 0.05, dashed *p* < 0.05, dot-dash *p* < 0.01, dotted *p* < 0.001. Age, body mass index (BMI), diet, activity status, and time of day when blood was collected were used as covariates in this moderation model.

The GEE model for PSQI scores did not show a significant main effect of time; however, there was a significant group effect, group-by-time interaction, and group-by-time-by-cytokine concentration interaction (Table 2). Results and tests of simple effects for the group-by-time interaction can be found in Supplementary Figure 5. In the cannabis non-use participants, there were no significant changes in sleep quality over time regardless of cytokine concentration (*p*’s > 0.05). At low cytokine concentrations, there were trends for PSQI scores to decrease over time in the THC + CBD (difference = −1.73, SE = 0.6, *p* = 0.05) and CBD (difference = −1.81, SE = 0.7, *p* = 0.08) groups, but not the THC (difference = 0.09, SE = 0.7, *p* = 1.0) group. All three cannabis use groups experienced significant decreases in PSQI scores at average (THC + CBD: difference = −1.83, SE = 0.5, *p* = 0.002; THC: difference = −0.88, SE = 0.3, *p* = 0.04; CBD: difference = −1.95, SE = 0.5, *p* < 0.001) and high cytokine concentrations (THC + CBD: difference = −1.92, SE = 0.4, *p* < 0.001; THC: difference = −1.84, SE = 0.5, *p* = 0.006; CBD: difference = −2.09, SE = 0.4, *p* < 0.001) (Figure 3).

Because there were no significant changes in PSQI scores over time at any baseline cytokine concentration in participants who did not use cannabis, comparisons of the rate of change over time were only performed between the three cannabis use groups. At low baseline cytokine concentrations, it was observed that PSQI scores for the THC + CBD and CBD groups decreased at a significantly greater rate compared to the THC group (THC + CBD: difference = −1.82, SE = 0.9, *p* = 0.04; CBD: difference = −1.9, SE = 0.9, *p* = 0.04). There were also trends for PSQI scores of both the THC + CBD and CBD groups to decrease at a greater rate when compared to the THC group (THC + CBD: difference = −0.95, SE = 0.6, *p* = 0.09; CBD: difference = −1.08, SE = 0.6, *p* = 0.06). There were no significant differences in the rate of change in PSQI scores between any of the cannabis groups at high baseline cytokine concentrations.

### Discussion

This study presents novel findings regarding the role of cytokines in moderating the effects of cannabis use on negative affect and subjective sleep quality in individuals with mild or greater anxiety. In general, our first hypothesis was not supported, as the use of cannabis was not associated with changes in cytokine concentration from Baseline to Week-4 of the study. However, when baseline inflammatory status was considered in the analysis as a moderator rather than a mediator, distinct patterns of change by cannabis use/non-use groups emerged. Specifically, the THC + CBD and CBD groups showed significant decreases in DASS total scores at all baseline cytokine concentrations. Decreases in DASS total scores were less consistent across cytokine concentrations in the THC and cannabis non-use groups.

In the THC group, significant decreases in negative affect were only observed at average baseline cytokine concentrations. Similarly, the cannabis non-use group only observed significant decreases at average and high baseline cytokine concentrations. The inconsistent effects in the THC and cannabis non-use groups could be cytokine-dependent or partly explained by natural regression to the mean as a result of fluctuations in negative affect over time. Contrasted to the consistent decreases in negative affect observed in the THC + CBD and CBD groups, this suggests that cannabis products containing higher amounts of CBD are more effective at reducing negative affect in individuals with mild or greater anxiety regardless of their baseline inflammatory status. This finding is further supported when comparing the rate of change in DASS total scores of these groups from Baseline to Week-4. Participants in the CBD group experienced a greater rate of decrease in DASS total scores at all (low, average, and high) baseline cytokine concentrations when compared to participants who did not use cannabis.

The more nuanced aspects of the DASS-21, via the three subscales, suggest that changes in the combination of depression, anxiety, and stress as measured by the DASS total score are primarily driven by changes in depression and anxiety, not stress. Our hypothesis of products containing higher amounts of CBD being associated with greater decreases in negative affect over time was further supported by observing that the CBD group experienced significant improvements in depression and anxiety at all Baseline cytokine concentrations. These findings indicate that THC-dominant products impacted affective states more selectively, only producing significant decreases in depression and anxiety at average cytokine concentrations. Interestingly, there were no significant changes in depression and anxiety among non-use participants regardless of cytokine concentration, suggesting that the significant changes in DASS total for these participants are not attributable to changes in any specific affective component. Although further exploration is needed, these findings suggest that THC-dominant products have limited impact on affective symptoms when coupled with extreme levels of inflammation (low or high). Comparatively, products that are CBD-dominant or contain equal parts THC and CBD have more consistent application across a wide range of inflammatory states.

To our knowledge, cytokine concentration has generally not been explored in the relationship between cannabis chemovar and negative affect. While results do not support the first proposed mechanism of this study (mediation of the impact of cannabis on behavioral health by changes in circulating cytokines), they do support the second. Specifically, the data show that baseline inflammatory status plays a role in moderating the response to cannabis on measures of negative affect. This moderation may be driven by cannabis’ previously reported acute effects on the release of inflammatory cytokines. Compared to THC, CBD has been observed to have more potent suppression of inflammatory cytokine release that is independent of CB1 and CB2 activation (Kozela et al., 2010). Rather, this immunosupression appears to be a result of CBD reducing the activity of the NF-kappaB pathway and up-regulating the STAT3 transcription factor in immune cells (Kozela et al., 2010). While not directly tested in the current paper, this anti-inflammatory activity may account for the more consistent reductions in negative affect across inflammatory states observed in the THC + CBD and CBD groups compared to the limited reductions in the THC group. Given that fluctuations in inflammatory status are associated with changes in negative affect (Hoge et al., 2009; Vieira et al., 2010; Gualtieri et al., 2020), the use of CBD may promote an immunomodulatory effect, limiting severe deviations from a person’s regular inflammatory state and suppressing inflammatory-driven deviations in negative affect. Though speculative, this theory warrants further exploration.

An explanation for the lack of cannabis-mediated immunosuppressive effects observed in the present study may be the result of previously published pre-clinical studies not utilizing THC or CBD concentrations reflective of normal blood concentrations reached post-acute flower use. A previous study from our laboratory observed peak blood THC and CBD concentrations 15 min post-flower-use of 74 ng/mL and 32 ng/mL respectively (Bidwell et al., 2024). Using the molecular weights of THC (314.45 g/mol) and CBD (314.47 g/mol) these observed values would translate to 0.02 μM THC and 0.01 μM CBD. Pre-clinical stimulations observing the immunosuppressive effects of THC and CBD have used molarities of 1–10 μM (Kozela et al., 2010). The lack of cannabis-mediated immunosuppressive effects reported in the present study may be the result of pharmacological differences between pre-clinical and clinical research. Yet, based on our findings, it is clear that inflammatory status plays a role in moderating the anxiolytic effects of cannabis. Future research in this area should focus on biobehavioral approaches to further assess the underlying relationship between cannabis, anxiety, and inflammation.

Regarding sleep quality, there were no significant improvements observed over time in participants who were not using any cannabis products regardless of their Baseline inflammatory state. In the groups using cannabis, both Baseline inflammatory status and cannabis product type heavily influenced improvements in sleep quality over time. Although significant improvements in sleep quality were observed in all three cannabis use groups at average and high baseline cytokine concentrations, there was a trend for the two chemovar groups containing greater amounts of CBD (THC + CBD and CBD) to observe greater rates of improvement in their sleep quality when compared to the THC group at average baseline cytokine concentrations. These findings suggest that the improvements in subjective sleep quality over time are associated with cannabis use, are generally more impactful in those with average or greater inflammation, and are more pronounced in products with higher amounts of CBD. This supports our hypothesis and is consistent with previous literature observing improvements in sleep quality associated with the use of cannabis products higher in CBD over administration periods ranging from 4 to 8 weeks (Bidwell et al., 2023; Dahlgren et al., 2022; Kisiolek et al., 2023). A novel contribution of the present analysis is that it is the first to explore the impact of baseline inflammatory state as a moderator of cannabis’ effects on sleep quality in individuals with anxiety. Further, our report of limited impacts by any cannabis product to improve subjective sleep quality in participants with low levels of inflammation may help to explain the varying effects of cannabis on sleep reported in current literature.

Previous research has provided inconclusive evidence regarding the effects of THC on negative affect and sleep. Some studies have observed improvements in sleep quality (Moreno-Sanz et al., 2022; Ueberall et al., 2022) and anxiety symptoms (Bidwell et al., 2024; Moreno-Sanz et al., 2022) with the use of THC-dominant cannabis products. Others have reported no changes in sleep quality (Whitehurst et al., 2015; Nicholson et al., 2004; Spanagel and Bilbao, 2021) or anxiety (Matheson et al., 2020) with the use of THC-dominant products. There are even studies that have reported the use of THC being associated with worsened anxiety (Hutten et al., 2022) and sleep quality (Winiger et al., 2022; Ly and Gehricke, 2013; Edwards and Filbey, 2021). Our findings suggest inflammatory status as a potential explanation for some of the variability in outcomes. It is recommended that future research exploring health outcomes related to cannabis use continue to assess other individual differences, like inflammation, as moderators of these effects.

The present study had both strengths and weaknesses that should be noted. Cannabis abstinence (non-use group) and chemovar adherence (THC + CBD, THC, and CBD groups) in the non-use group were verified via blood THC and CBD concentrations at the Week-4 visit. The naturalistic, *ad libitum* administration of cannabis flower products purchased on the legal market strengthens the external validity of our findings. Participants were instructed to use their cannabis product as much or as little as they liked and were not instructed to use their product at a specific time or dose each day such that use in this study mirrored their typical use. That said, more research is needed to further explore if the use of cannabis products at a specific time of day (i.e., morning or night) would promote similar effects for the outcomes of negative affect and sleep.

Despite the strength of the naturalistic design of the study, the lack of a placebo control condition should be noted as it may have influenced our findings. Specifically, participants who believe cannabis is beneficial may have reported improvements due to placebo effects rather than true pharmacological effects. Unfortunately, given that cannabis is classified as a Schedule 1 substance in the US, there are federal restrictions that make the use of a placebo condition infeasible, at least in the context of the current study. In particular, researchers are prohibited from administering controlled doses of legal-market cannabis to study participants. Further, dispensaries and hemp shops are required to report the cannabinoid content on the product label such that participants will always be aware of the type of product they are using. However, future research should endeavor to employ innovative research designs that implement placebo control conditions as much as possible within the limits of these federal regulations.

Participants assigned to the cannabis use groups in this study used their products on average every other day, and thus these findings are not reflective of daily or occasional cannabis use. Further, our findings are limited to flower-based cannabis products, and it is unclear if the same pattern of findings would extend to other methods of cannabis use (i.e., edibles, tinctures, and concentrates). It should also be noted that frequency of cannabis use or other objective measures of cannabis use (i.e., blood THC and CBD) were not able to be included in the statistical models due to the cannabis non-use group having 0’s for these values across all participants. Future research should further explore the impact of cannabinoid dose and frequency of use in addition to other methods of cannabis use and their associated effects on negative affect and sleep quality. Further, due to the relatively short observational period of the study (4 weeks), future studies should explore whether these effects are preserved over a longer duration of time or reversed with the cessation of cannabis product use. Finally, the authors would like to note that these patterns were explored in an anxious population that captured a broad range of self-reported anxiety symptoms (mild to severe). In the future, these relationships should also be explored in DSM-5 defined clinical populations with more severe symptoms of anxiety.

In conclusion, this study presents novel data on the impact of Baseline inflammatory status moderating changes in negative affect and sleep quality in individuals with mild or greater anxiety. Over 4 weeks, it was observed that cannabis flower products containing moderate to high amounts of CBD and relatively low THC demonstrated consistent significant decreases in negative affect (DASS total) regardless of baseline inflammatory status. Significant consistent reductions in negative affect were attenuated in both participants not using any cannabis products and in those using products with relatively high THC with significant reductions varying by baseline inflammatory status. There were no significant improvements in subjective sleep quality observed in participants who did not use cannabis products. Cannabis use was associated with significant improvements in sleep quality in those with average and high, baseline cytokine concentrations. There is also preliminary evidence that improvements in both negative affect and sleep quality may be more pronounced in participants using cannabis products with moderate to high amounts of CBD when compared to participants using products relatively high in THC. These findings on cytokine-dependent effects may shed light on the current equivocal evidence regarding cannabis use for negative affect and sleep.

## Data Availability

The raw data supporting the conclusions of this article will be made available by the authors, without undue reservation.

## References

[B1] Abd El-Kader S. M. Al-Jiffri O. H. (2019). Aerobic exercise modulates cytokine profile and sleep quality in elderly. *Afr. Health Sci.* 19 2198–2207. 10.4314/ahs.v19i2.45 31656505 PMC6794533

[B2] Adjoian T. Firestone M. Eisenhower D. Yi S. (2016). Validation of self-rated overall diet quality by healthy eating index-2010 score among New York City adults, 2013. *Prev. Med. Rep.* 3 127–131. 10.1016/j.pmedr.2016.01.001 26844200 PMC4733090

[B3] Bai S. Guo W. Feng Y. Deng H. Li G. Nie H. (2020). Efficacy and safety of anti-inflammatory agents for the treatment of major depressive disorder: A systematic review and meta-analysis of randomised controlled trials. *J. Neurol. Neurosurg. Psychiatry* 91 21–32. 10.1136/jnnp-2019-320912 31658959

[B4] Bauer D. J. Curran P. J. (2005). Probing interactions in fixed and multilevel regression: Inferential and graphical techniques. *Multivar. Behav. Res.* 40 373–400. 10.1207/s15327906mbr4003_5 26794689

[B5] Berk M. Williams L. Jacka F. O’Neil A. Pasco J. Moylan S. (2013). So depression is an inflammatory disease, but where does the inflammation come from? *BMC Med.* 11:200. 10.1186/1741-7015-11-200 24228900 PMC3846682

[B6] Bidwell L. Martin-Willett R. Skrzynski C. Lisano J. Ortiz Torres M. Giordano G. (2024). Acute and extended anxiolytic effects of cannabidiol in cannabis flower: A quasi-experimental ad libitum use study. *Cannabis Cannabinoid Res.* 9 1015–1027. 10.1089/can.2023.0187 38252547 PMC11392455

[B7] Bidwell L. Sznitman S. Martin-Willett R. Hitchcock L. (2023). Daily associations with cannabis use and sleep quality in anxious cannabis users. *Behav. Sleep Med.* 22 150–167. 10.1080/15402002.2023.2217969 37255232 PMC10687319

[B8] Buysse D. Reynolds C. Monk T. Berman S. Kupfer D. (1989). The Pittsburgh sleep quality index: A new instrument for psychiatric practice and research. *Psychiatry Res.* 28 193–213. 10.1016/0165-1781(89)90047-4 2748771

[B9] Callahan T. Caldwell Hooper A. Thayer R. Magnan R. Bryan A. (2013). Relationships between marijuana dependence and condom use intentions and behavior among justice-involved adolescents. *AIDS Behav.* 17 2715–2724. 10.1007/s10461-013-0417-0 23370834 PMC3676463

[B10] Carey V. J. Lumley T. S. Moler C. Ripley B. (2023). *Generalized estimation equation solver. 4.13-23*.

[B11] Cohen J. (2013). *Statistical power analysis for the behavioral sciences.* New York, NY: Routledge.

[B12] Coksevim N. Durmus D. Kuru O. (2018). Effects of global postural reeducation exercise and anti-TNF treatments on disease activity, function, fatigue, mobility, sleep quality and depression in patients with active ankylosing spondylitis: A prospective follow-up study. *J. Back Musculoskelet. Rehabil.* 31 1005–1012. 10.3233/BMR-170901 30412478

[B13] Dahlgren M. Lambros A. Smith R. Sagar K. El-Abboud C. Gruber S. (2022). Clinical and cognitive improvement following full-spectrum, high-cannabidiol treatment for anxiety: Open-label data from a two-stage, phase 2 clinical trial. *Commun. Med.* 2:139. 10.1038/s43856-022-00202-8 36352103 PMC9628346

[B14] Davis J. Knutson K. Strausbauch M. Crowson C. Therneau T. Wettstein P. (2010). Analysis of complex biomarkers for human immune-mediated disorders based on cytokine responsiveness of peripheral blood cells. *J. Immunol.* 184 7297–7304. 10.4049/jimmunol.0904180 20495063 PMC2882518

[B15] Edwards D. Filbey F. M. (2021). Are sweet dreams made of these? Understanding the relationship between sleep and cannabis use. *Cannabis Cannabinoid Res.* 6 462–473. 10.1089/can.2020.0174 34143657 PMC8713269

[B16] Faridhosseini F. Sadeghi R. Farid L. Pourgholami M. (2014). Celecoxib: A new augmentation strategy for depressive mood episodes. A systematic review and meta-analysis of randomized placebo-controlled trials. *Hum. Psychopharmacol.* 29 216–223. 10.1002/hup.2401 24911574

[B17] Faul F. Erdfelder E. Lang A. Buchner A. (2007). G*Power 3: A flexible statistical power analysis program for the social, behavioral, and biomedical sciences. *Behav. Res. Methods* 39 175–191. 10.3758/bf03193146 17695343

[B18] Gualtieri P. Marchetti M. Cioccoloni G. De Lorenzo A. Romano L. Cammarano A. (2020). Psychobiotics regulate the anxiety symptoms in carriers of allele A of IL-1 β gene: A randomized, placebo-controlled clinical trial. *Mediat. Inflamm.* 2020:2346126. 10.1155/2020/2346126 32377159 PMC7199572

[B19] Harris P. Taylor R. Thielke R. Payne J. Gonzalez N. Conde J. (2009). Research electronic data capture (REDCap)–a metadata-driven methodology and workflow process for providing translational research informatics support. *J. Biomed. Inform.* 42 377–381. 10.1016/j.jbi.2008.08.010 18929686 PMC2700030

[B20] Henry J. D. Crawford J. R. (2005). The short-form version of the depression anxiety stress scales (DASS-21): Construct validity and normative data in a large non-clinical sample. *Br. J. Clin. Psychol.* 44 227–239. 10.1348/014466505X29657 16004657

[B21] Henshaw F. Dewsbury L. Lim C. Steiner G. (2021). The effects of cannabinoids on pro- and anti-inflammatory cytokines: A systematic review of in vivo studies. *Cannabis Cannabinoid Res.* 6 177–195. 10.1089/can.2020.0105 33998900 PMC8266561

[B22] Hoge E. Brandstetter K. Moshier S. Pollack M. Wong K. Simon N. (2009). Broad spectrum of cytokine abnormalities in panic disorder and posttraumatic stress disorder. *Depress. Anxiety* 26 447–455. 10.1002/da.20564 19319993

[B23] Hutten N. Arkell T. Vinckenbosch F. Schepers J. Kevin R. Theunissen E. (2022). Cannabis containing equivalent concentrations of delta-9-tetrahydrocannabinol (THC) and cannabidiol (CBD) induces less state anxiety than THC-dominant cannabis. *Psychopharmacology (Berl)* 239 3731–3741. 10.1007/s00213-022-06248-9 36227352 PMC9584997

[B24] Johnson E. O. Roth T. Breslau N. (2006). The association of insomnia with anxiety disorders and depression: Exploration of the direction of risk. *J. Psychiatr. Res.* 40 700–708. 10.1016/j.jpsychires.2006.07.008 16978649

[B25] Johnson S. Hoffart A. Tilden T. Toft H. Neupane S. Lien L. (2021). Circulating cytokine levels in the treatment of comorbid anxiety disorders. *Acta Neuropsychiatr.* 33 65–71. 10.1017/neu.2020.38 33109296

[B26] Kiernan M. Schoffman D. Lee K. Brown S. Fair J. Perri M. (2013). The stanford leisure-time activity categorical item (L-Cat): A single categorical item sensitive to physical activity changes in overweight/obese women. *Int. J. Obes.* 37 1597–1602. 10.1038/ijo.2013.36 23588625 PMC4731089

[B27] Kisiolek J. Flores V. Ramani A. Butler B. Haughian J. Stewart L. (2023). Eight weeks of daily cannabidiol supplementation improves sleep quality and immune cell cytotoxicity. *Nutrients* 15:4173. 10.3390/nu15194173 37836465 PMC10574483

[B28] Klawitter J. Sempio C. Mörlein S. De Bloois E. Klepacki J. Henthorn T. (2017). An Atmospheric pressure chemical ionization MS/MS assay using online extraction for the analysis of 11 cannabinoids and metabolites in human plasma and urine. *Ther. Drug Monit.* 39 556–564. 10.1097/FTD.0000000000000427 28640062 PMC5600652

[B29] Kozela E. Pietr M. Juknat A. Rimmerman N. Levy R. Vogel Z. (2010). Cannabinoids Delta(9)-tetrahydrocannabinol and cannabidiol differentially inhibit the lipopolysaccharide-activated NF-kappaB and interferon-beta/STAT proinflammatory pathways in BV-2 microglial cells. *J. Biol. Chem.* 285 1616–1626. 10.1074/jbc.M109.069294 19910459 PMC2804319

[B30] Lee C. Giuliani F. (2019). The role of inflammation in depression and fatigue. *Front. Immunol.* 10:1696. 10.3389/fimmu.2019.01696 31379879 PMC6658985

[B31] Lee H. Hong J. Kim J. Kim D. Jang S. Han S. (2021). Effects of probiotic NVP-1704 on mental health and sleep in healthy adults: An 8-week randomized, double-blind, placebo-controlled trial. *Nutrients* 13:2660. 10.3390/nu13082660 34444820 PMC8398773

[B32] Lenth R. (2023). *emmeans: Estimated marginal means, aka least-squares means. 1.8.5.*

[B33] Lozano B. E. Stephens R. S. Roffman R. A. (2006). Abstinence and moderate use goals in the treatment of marijuana dependence. *Addiction* 101 1589–1597. 10.1111/j.1360-0443.2006.01609.x 17034438

[B34] Lüdecke D. (2023). *sjPlot: Data visualization for statistics in social science. 2.8.13.*

[B35] Ly C. Gehricke J. (2013). Marijuana use is associated with inattention in men and sleep quality in women with attention-deficit/hyperactivity disorder: A preliminary study. *Psychiatry Res.* 210 1310–1312. 10.1016/j.psychres.2013.08.003 23993465 PMC3840066

[B36] Mahmud S. Mohsin M. Dewan M. N. Muyeed A. (2023). The global prevalence of depression, anxiety, stress, and insomnia among general population during COVID-19 pandemic: A systematic review and meta-analysis. *Trends Psychol.* 31 143–170. 10.1007/s43076-021-00116-9 40477944 PMC8726528

[B37] Manigault A. Ganz P. Irwin M. Cole S. Kuhlman K. Bower J. (2021). Moderators of inflammation-related depression: A prospective study of breast cancer survivors. *Transl. Psychiatry* 11 615. 10.1038/s41398-021-01744-6 34873150 PMC8648787

[B38] Martin-Willett R. Helmuth T. Abraha M. Bryan A. Hitchcock L. Lee K. (2020). Validation of a multisubstance online timeline followback assessment. *Brain Behav.* 10:e01486. 10.1002/brb3.1486 31793226 PMC6955818

[B39] Mason E. Grierson A. Sie A. Sharrock M. Li I. Chen A. (2023). Co-occurring insomnia and anxiety: A randomized controlled trial of internet cognitive behavioral therapy for insomnia versus internet cognitive behavioral therapy for anxiety. *Sleep* 46:zsac205. 10.1093/sleep/zsac205 36041459

[B40] Matheson J. Sproule B. Di Ciano P. Fares A. Le Foll B. Mann R. (2020). Sex differences in the acute effects of smoked cannabis: Evidence from a human laboratory study of young adults. *Psychopharmacology (Berl)* 237 305–316. 10.1007/s00213-019-05369-y 31637452

[B41] Moreno-Sanz G. Madiedo A. Lynskey M. Brown M. (2022). “Flower power”: Controlled inhalation of THC-predominant cannabis flos improves health-related quality of life and symptoms of chronic pain and anxiety in eligible UK patients. *Biomedicines* 10:2576. 10.3390/biomedicines10102576 36289837 PMC9599241

[B42] Morin C. Bertisch S. Pelayo R. Watson N. Winkelman J. Zee P. (2023). What should be the focus of treatment when insomnia disorder is comorbid with depression or anxiety disorder? *J. Clin. Med.* 12:1975. 10.3390/jcm12051975 36902762 PMC10004168

[B43] Nicholson A. N. Turner C. Stone B. M. Robson P. J. (2004). Effect of delta-9-tetrahydrocannabinol and cannabidiol on nocturnal sleep and early-morning behavior in young adults. *J. Clin. Psychopharmacol.* 24 305–313. 10.1097/01.jcp.0000125688.05091.8f 15118485

[B44] Papadimitriou G. N. Linkowski P. (2005). Sleep disturbance in anxiety disorders. *Int. Rev. Psychiatry* 17 229–236. 10.1080/09540260500104524 16194794

[B45] R Core Team (2020). *R: A language and environment for statistical computing.* Vienna: R Core Team.

[B46] Reinarman C. Nunberg H. Lanthier F. Heddleston T. (2011). Who are medical marijuana patients? Population characteristics from nine California assessment clinics. *J. Psychoact. Drugs* 43 128–135. 10.1080/02791072.2011.587700 21858958

[B47] Ried K. Tamanna T. Matthews S. Sali A. (2023). Medicinal cannabis improves sleep in adults with insomnia: A randomised double-blind placebo-controlled crossover study. *J. Sleep Res.* 32 e13793. 10.1111/jsr.13793 36539991

[B48] Rizopoulos D. (2022). *Latent trait models under IRT. 1.2-0.*

[B49] Spanagel R. Bilbao A. (2021). Approved cannabinoids for medical purposes – comparative systematic review and meta-analysis for sleep and appetite. *Neuropharmacology* 196:108680. 10.1016/j.neuropharm.2021.108680 34181977

[B50] Spitzer R. L. Kroenke K. Williams J. B. W. Löwe B. (2006). A brief measure for assessing generalized anxiety disorder: The GAD-7. *Arch. Intern. Med.* 166 1092–1097. 10.1001/archinte.166.10.1092 16717171

[B51] Subar A. Thompson F. Kipnis V. Midthune D. Hurwitz P. McNutt S. (2001). Comparative validation of the Block, Willett, and national cancer institute food frequency questionnaires: The eating at America’s table study. *Am. J. Epidemiol.* 154 1089–1099. 10.1093/aje/154.12.1089 11744511

[B52] Tervo-Clemmens B. Schmitt W. Wheeler G. Cooke M. Schuster R. Hickey S. (2023). Cannabis use and sleep quality in daily life: An electronic daily diary study of adults starting cannabis for health concerns. *Drug Alcohol Depend.* 243:109760. 10.1016/j.drugalcdep.2022.109760 36638745 PMC10015315

[B53] Ueberall M. Horlemann J. Schuermann N. Kalaba M. Ware M. (2022). Effectiveness and tolerability of dronabinol use in patients with chronic pain: A retrospective analysis of 12-week open-label real-world data provided by the german pain e-registry. *Pain Med.* 23 1409–1422. 10.1093/pm/pnac010 35104881 PMC9340619

[B54] Vieira M. Ferreira T. Pacheco P. Barros P. Almeida C. Araújo-Lima C. (2010). Enhanced Th17 phenotype in individuals with generalized anxiety disorder. *J. Neuroimmunol.* 229 212–218. 10.1016/j.jneuroim.2010.07.018 20709414

[B55] Walsh J. Maddison K. Rankin T. Murray K. McArdle N. Ree M. (2021). Treating insomnia symptoms with medicinal cannabis: A randomized, crossover trial of the efficacy of a cannabinoid medicine compared with placebo. *Sleep* 44:zsab149. 10.1093/sleep/zsab149 34115851 PMC8598183

[B56] Whitehurst L. Fogler K. Hall K. Hartmann M. Dyche J. (2015). The effects of chronic marijuana use on circadian entrainment. *Chronobiol. Int.* 32 561–567. 10.3109/07420528.2015.1004078 25801606

[B57] Wickham H. François R. Henry L. Müller K. Vaughan D. (2023). *dplyr: A grammar of data manipulation. 1.1.2.*

[B58] Wickham H. Navarro D. Pedersen T. L. (2024). *ggplot2: Create elegant data visualisations using the grammar of graphics. 3.4.1.*

[B59] Winiger E. Ma M. Brooks-Russell A. (2022). Novel methods of cannabis use and lower sleep duration among high school students. *Cannabis* 5 66–73. 10.26828/cannabis/2022.02.006 37287665 PMC10212238

[B60] Zhang Y. Hedo R. Rivera A. Rull R. Richardson S. Tu X. (2019). Post hoc power analysis: Is it an informative and meaningful analysis? *Gen. Psychiatr.* 32:e100069. 10.1136/gpsych-2019-100069 31552383 PMC6738696

