# Supplementary material for: Inflammatory state moderates response to cannabis on negative affect and sleep quality in individuals with anxiety
Source: Front Behav Neurosci. 2025 Jul 1;19:1549311. doi: 10.3389/fnbeh.2025.1549311 (PMC12259704; doi:10.3389/fnbeh.2025.1549311)
Supplement: Supplementary file 1 [file Table_1.docx]

**Supplemental Figure 1: Participant Consort**

**
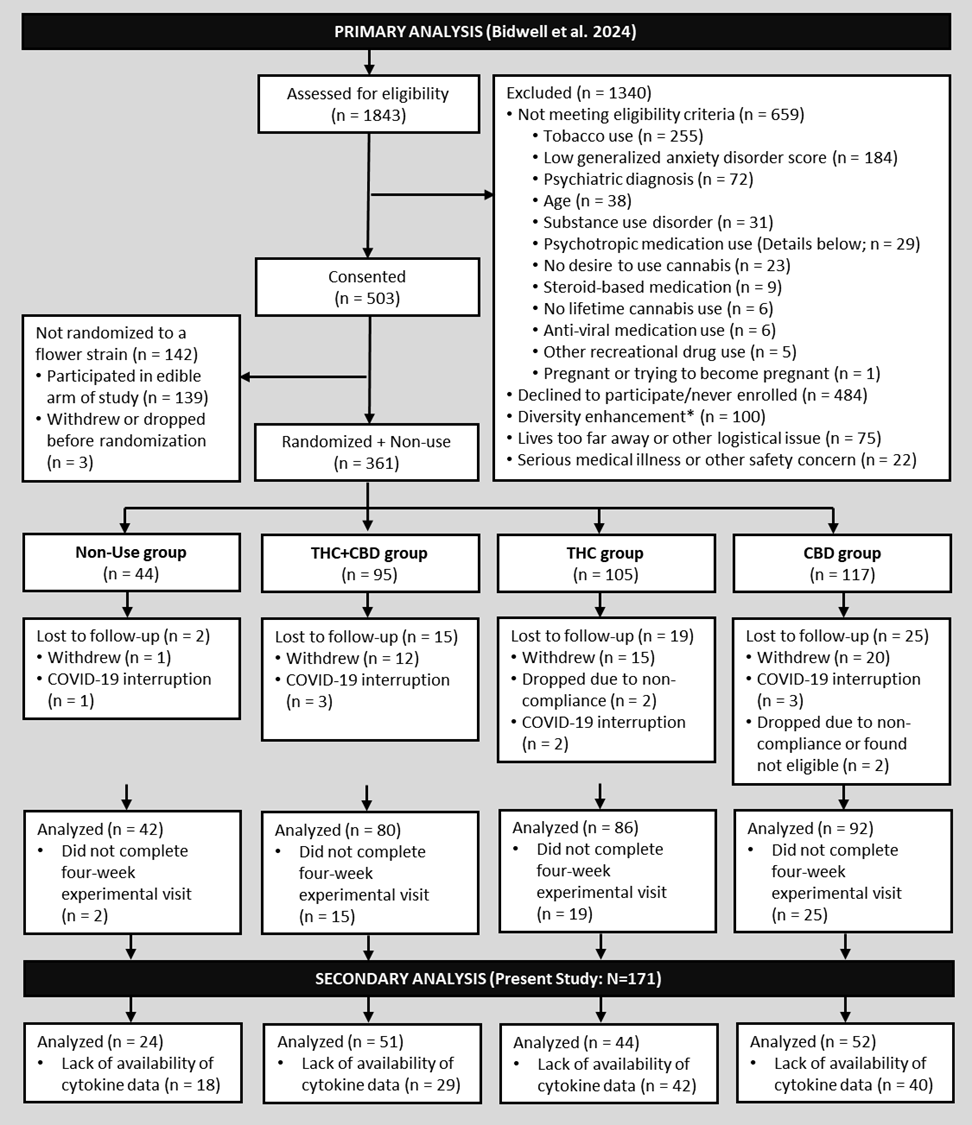
**

*Note.* Participants included in this secondary analysis (present study) were pulled from the primary analysis presented in Bidwell et al. 2024 based on the availability of cytokine data.

**Supplemental Figure 2. Correlation Table**


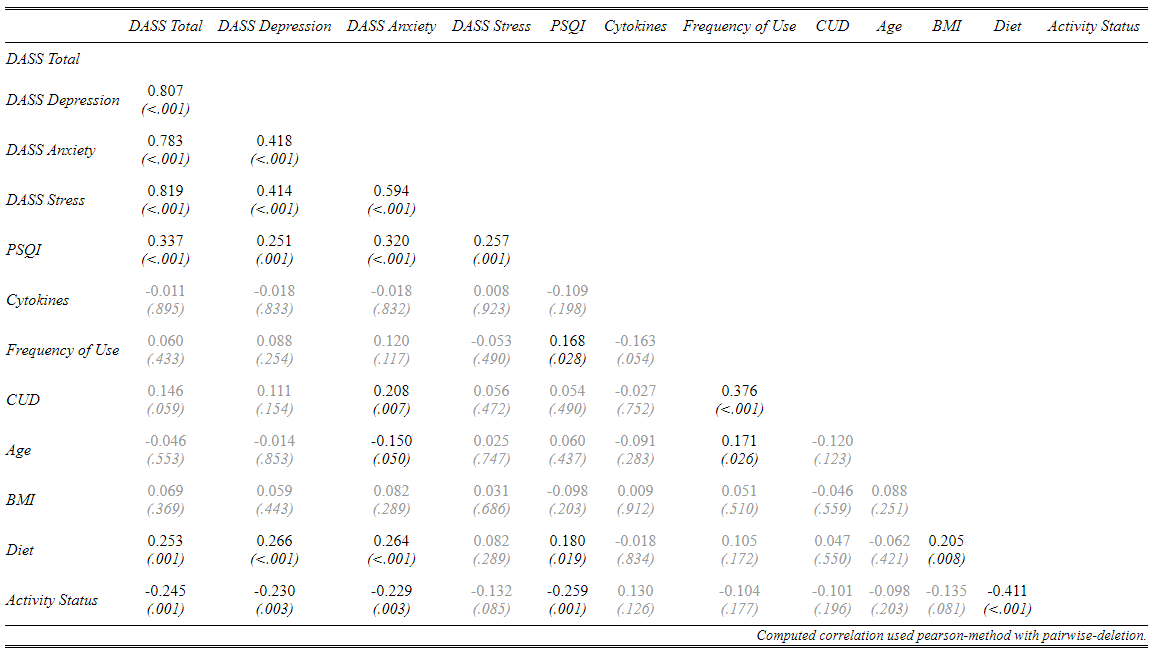


*Note.* Depression, Anxiety, and Stress Scale (DASS), Pittsburg Sleep Quality Index (PSQI), Cannabis Use Disorder (CUD), and Body Mass Index (BMI). *p<0.05, **p<0.01, ***p<0.001

**Supplemental Figure 3. Blood Cannabinoid Concentration by Group at Week-4**


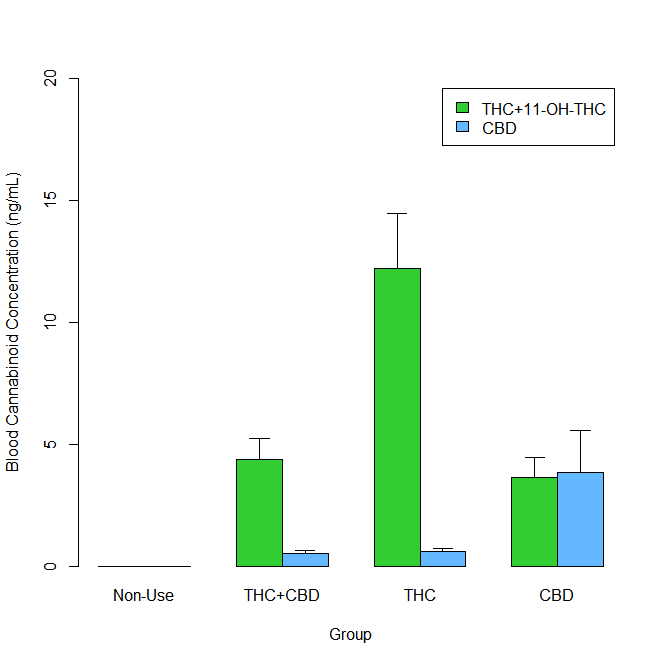


**Note.** Group means ± SE are depicted for Week-4 plasma concentrations of delta-9-tetrahydrocannabinol + 11-hydroxy-tetrahydrocannabinol (THC+11-OH-THC), and cannabidiol (CBD). Average THC+11-OH-THC was highest in the THC group, whereas average CBD was highest in the CBD group. No cannabinoids were detected in the Non-Use group.

**Supplemental Figure 4. Moderation Model of DASS Depression and Anxiety Subscale Scores by Group Controlling for Baseline Cytokine Concentrations**


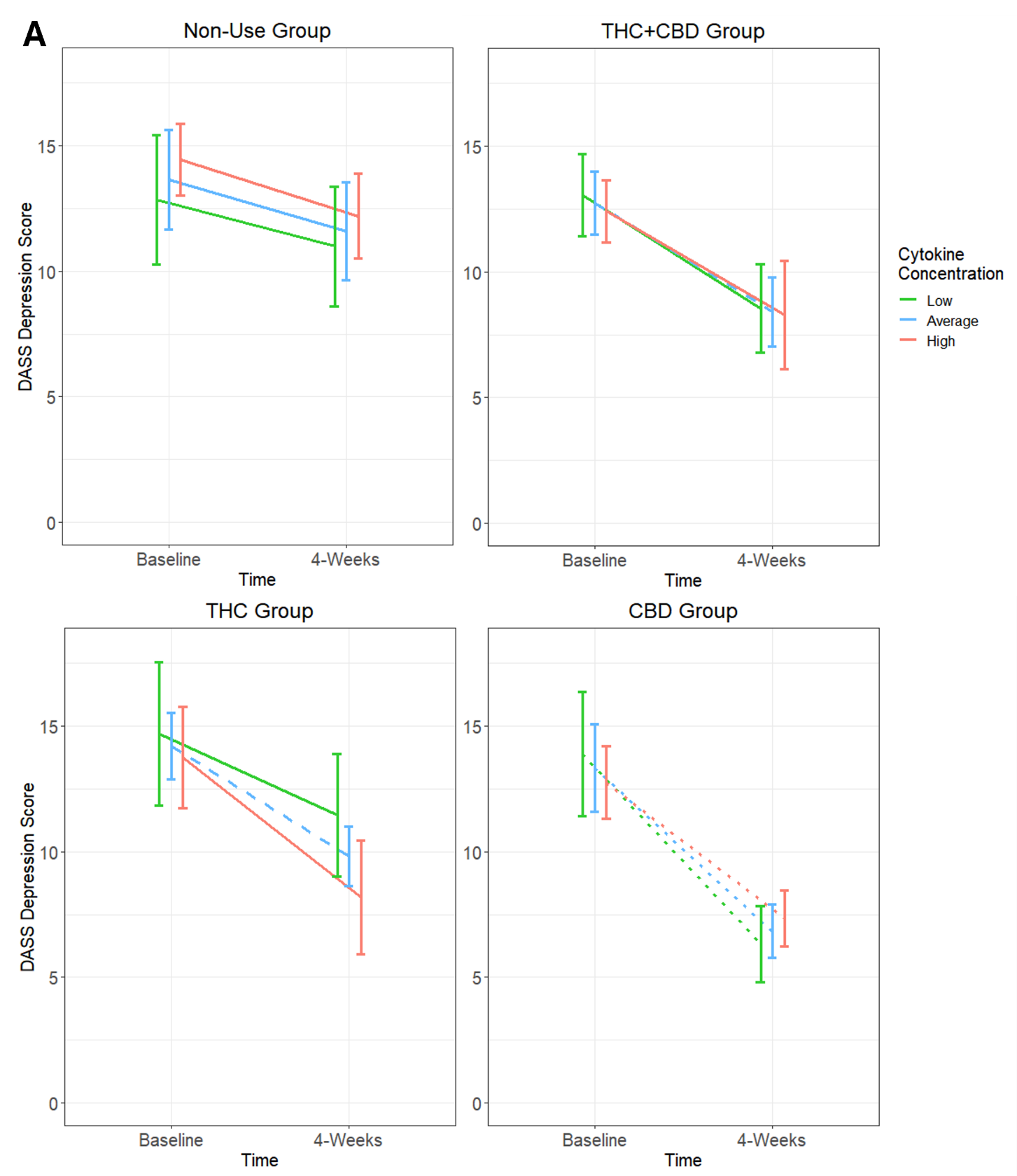


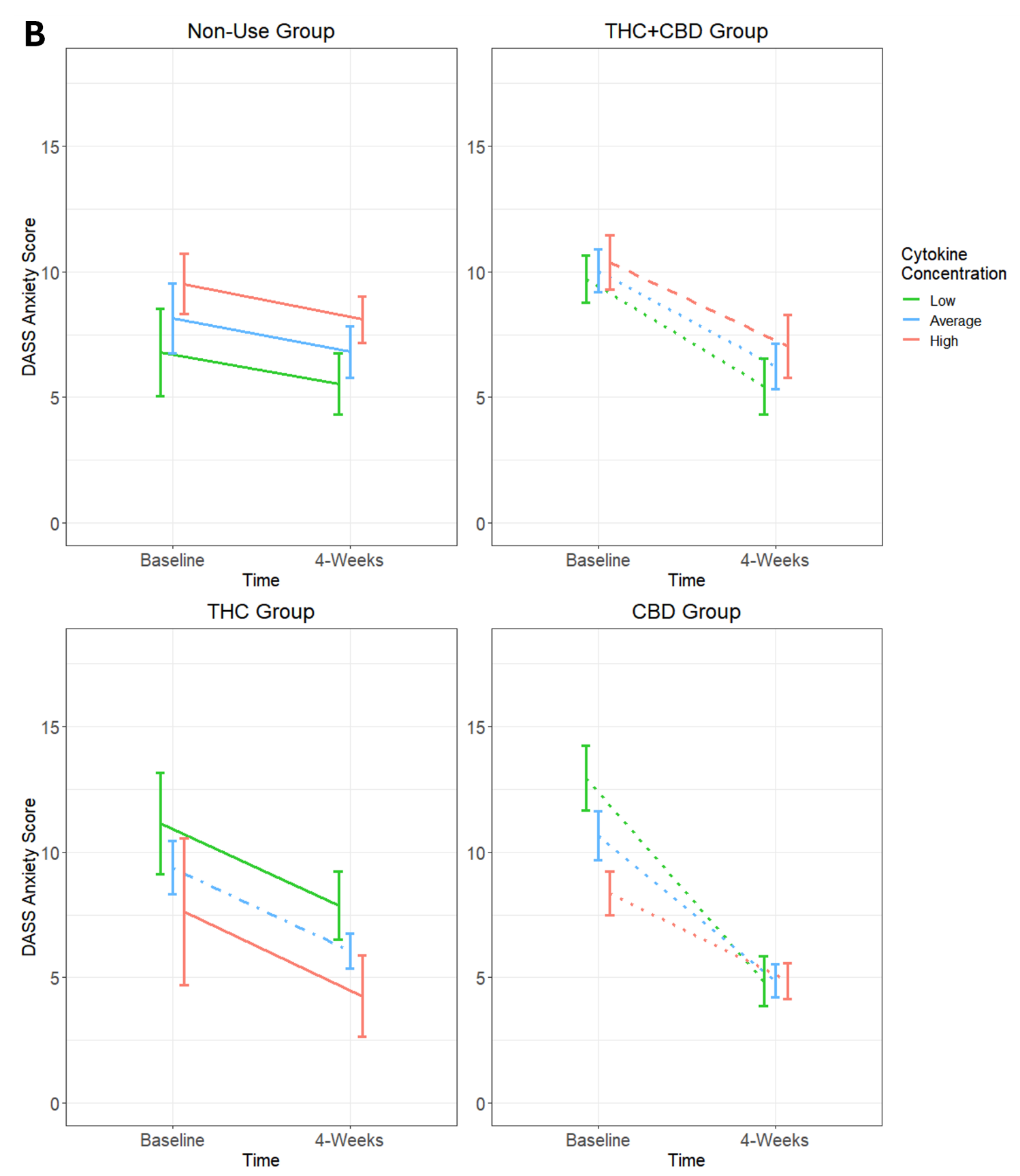


*Note.* A. DASS Depression. B. DASS Anxiety. Group estimated means ± SE are plotted for both sets of graphs. P-values of simple effects are represented by the following: solid p>0.05, dashed p<0.05, dot-dash p<0.01, dotted p<0.001. Age, body mass index (BMI), diet, activity status, and time of day when blood was collected were used as covariates in this moderation model.

**Supplemental Figure 5. Group by Time Effects for Pittsburg Sleep Quality Index Scores**


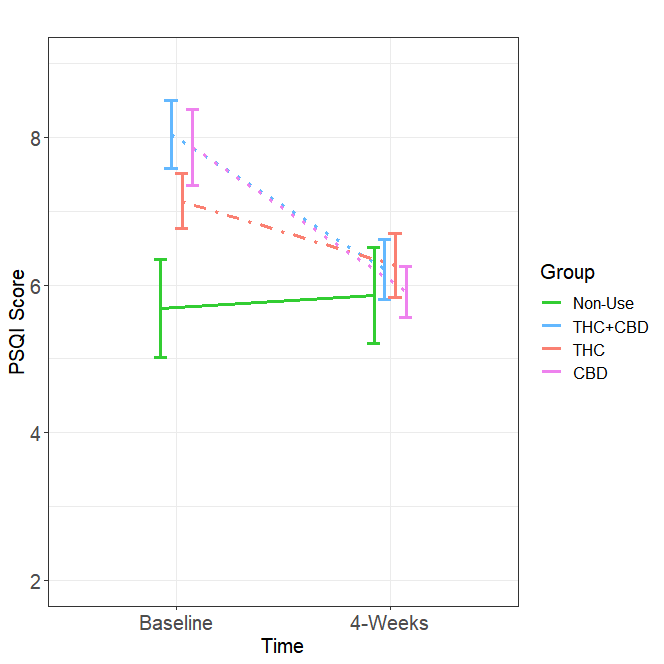


*Note.* Group estimated means ± SE are plotted. Simple effects by Group over Time: Non-Use (difference = 0.18, SE = 0.6, *p*=0.75), THC+CBD (difference = -1.83, SE = 0.5, *p*<0.001), THC (difference = -0.88, SE = 0.3, *p*=0.003), CBD (difference = -1.95, SE = 0.5, *p*<0.001). Age, body mass index (BMI), diet, activity status, and time of day when blood was collected were used as covariates in this moderation model.
